# Supplementary material for: Hematological abnormalities and associated factors among metabolic syndrome patients at the University of Gondar comprehensive specialized hospital, Northwest Ethiopia
Source: PLoS One. 2023 May 25;18(5):e0286163. doi: 10.1371/journal.pone.0286163 (PMC10212162; doi:10.1371/journal.pone.0286163)
Supplement: S1 File — (DOCX) [file pone.0286163.s001.docx]

**English version of the data collection tool (Questionnaire and checklist)**

| **Part I: Socio-demographic characteristics** | | |
| --- | --- | --- |
| **S.No** | **Variable** | **Response** |
| 101 | Gender | 1.Male 2.Female |
| 102 | Age | ________________ (in years) |
| 103 | Marital status | 1. Single 2. Married 3. Divorced 4. Widowed |
| 104 | Educational background | 1. No formal education 2. Primary education (1-8) 3. Secondary education (9-12) 4. Higher education |
| 105 | Residence | 1. Urban 2. Rural |
| 106 | Occupation | 1. Government employee 2. Non-government employee 3. Farmer 4. Self-employed 5. House wife 6. Retired 7. Unemployed 8. Others (specify) ________________ |

| **Part II: Patients’ behavioral characteristics** | | | **Skip** |
| --- | --- | --- | --- |
| **Tobacco use** | | | |
| 201 | Have you ever used any tobacco products such as cigarettes, cigars, pipes, shisha, snuff, or chewing tobacco? | 1. Yes 2. No | If no, skip to 204 |
| 202 | Do you currently use any tobacco products? | 1. Yes 2. No | If no, skip to 204 |
| 203 | On average, how many of the following tobacco products do you use each day? | 1. Manufactured cigarettes--------/day 2. Number of Shisha sessions--------/day 3. Other   (please specify): ---------/day | |
| **Alcohol consumption** | | | |
| 204 | Have you ever consumed any alcohol such as beer, wine, Tej, Tela, areki, korefe…) | 1. Yes 2. No | If no, skip to 207 |
| 205 | Do you currently drink alcohol? | 1. Yes 2. No | If no, skip to 207 |
| 206 | How frequently do you have at least one standard alcoholic drink in a week/month? | 1. 1-2 days per week 2. 3-4 days per week 3. 5-6 days per week 4. Daily 5. 1-3 days per month 6. Other(please specify) :------------- | |
| **Physical activity** | | | |
| 207 | Does your work involve a vigorous-intensity activity that causes large increases in breathing or heart rate like carrying or lifting heavy loads, digging, or construction work for at least 10 minutes continuously? | 1. Yes 2. No | If no, skip to 209 |
| 208 | In a typical week, on how many days do you do vigorous-intensity activities as part of your work? | Number of days-------- | |
| 209 | Does your work involve moderate-intensity activity that causes small increases in breathing or heart rate such as brisk walking, carrying light loads and, cleaning for at least 10 minutes continuously? | 1. Yes 2. No | If no, skip to 211 |
| 210 | In a typical week, on how many days do you do moderate-intensity activities as part of your work? | Number of days-------- | |
| 211 | Do you usually walk or use transport to get to and from places? | 1. I usually walk 2. I usually use transport | |
| 212 | How much time do you usually spend sitting or reclining on a typical day? | Hours ----------  Minutes ------------- | |

| **Part III: Patient’s dietary characteristics** | | | |
| --- | --- | --- | --- |
|  | In the past 7 days, how often have you eaten (in days); | Number of days | Never |
| 301 | Any food made from grains; injera, teff, millet, sorghum, maize, rice, wheat, bread, biscuits, or any other grain product or any food made from tubers potatoes, sweet potatoes, carrots, or other foods made from roots or tubers? |  |  |
| 302 | Any pulses (beans, lentils, peas, nuts)? |  |  |
| 303 | Any vegetables? |  |  |
| 304 | Any fruits? |  |  |
| 305 | Any meat: beef, lamb, goat, fish, chicken, liver, kidney, or other organ meats? |  |  |
| 306 | Any eggs? |  |  |
| 307 | Any dairy products; milk, cheese, yogurt? |  |  |
| 308 | Any sugar or honey? |  |  |
| 309 | Any oil, fat, or butter? |  |  |
| 310 | How often is salt added in cooking or preparing foods in your household? |  |  |
| 311 | How often do you eat processed foods? Such as packaged snacks, juices, canned foods, and soft drinks. |  |  |

| **Part IV: Patient’s anthropometric and clinical characteristics** | | | **Skip** |
| --- | --- | --- | --- |
| 401 | Height | ­­­­___________cm |  |
| 402 | Weight | ___________kg |  |
| 403 | BMI | ___________kg/m^2^ |  |
| 404 | Waist circumference | ___________cm |  |
| 405 | Blood Pressure (BP) | 1. Systolic Blood Pressure ___________mmHg 2. Diastolic Blood Pressure ___________mmHg |  |
| 406 | Duration since diagnosed with metabolic syndrome? | ----------Months---------Years |  |
| 407 | Is there anyone in your family who had a history of metabolic syndrome or its components, other than you? | 1. Yes 2. No | If no, skip to 409 |
| 408 | Which of the following metabolic syndrome components your family member have had?  (More than one option can be chosen) | 1. Hypertension 2. Diabetes mellitus 3. Dyslipidemia(increased triglyceride, reduced high density lipoprotein-cholesterol) 4. Abdominal obesity |  |
| 409 | Is the patient taking any medications for metabolic syndrome or its components prescribed by a doctor or other health worker? | 1. Yes 2. No | If no, skip to 501 |
| 410 | Which type of medications is the patient using? (More than one option can be chosen) | 1. Anti-hypertensive medication 2. Anti-diabetic medication 3. Anti-dislipidemic medication |  |

| **Part V: Patient’s laboratory data** | | | | | | | | |
| --- | --- | --- | --- | --- | --- | --- | --- | --- |
| **Biochemical and hematological measurements** | | | | | | | | |
| 501 | HDL-C(mg/dl) |  | 509 | MCH(pg) |  | 517 | MON% |  |
| 502 | TG(mg/dl) |  | 510 | MCHC(g/dl) |  | 518 | EOS% |  |
| 503 | FBG(mg/dl) |  | 511 | RDW (%) |  | 519 | BAS% |  |
| 504 | WBC(10^3^/*µ*l) |  | 512 | RDW-SD |  | 520 | NEU# |  |
| 505 | RBC(10^6^/*µ*l) |  | 513 | PLT(10^3^/*µ*l) |  | 521 | LYMP# |  |
| 506 | Hb(g/dl) |  | 514 | MPV(fl) |  | 522 | MON# |  |
| 507 | Hct(%) |  | 515 | NEU% |  | 523 | EOS# |  |
| 508 | MCV(fl) |  | 516 | LYMP% |  | 524 | BAS# |  |
| **Parasitological examination results** | | | | | | | | |
| 525 | Malaria | 1. H/P seen   If H/P seen Specify: ---------------------------------   1. No H/P seen | | | | | | |
| 527 | Intestinal parasite | 1. O/P seen   If O/P seen Specify: ------------------------------------   1. No O/P seen | | | | | | |

| **ተ.ቁጥር** | **ጥያቄ** | **ምላሽ** |
| --- | --- | --- |
| **ክፍል አንድ፡ ማህበራዊ የሰነህዝብ አወቃቀር ሁኔታን የሚያሳይ መጠይቅ** | | |
| 101 | ፆታ | \| 1. ወንድ 2. ሴት \|  \| \| --- \| --- \| |
| 102 | ዕድሜ | ________________ አመት |
| 103 | የጋብቻ ሁኔታ | \| 1. ያላገባ/ች 2. ያገባ/ች 3. የተፋቱ 4. የሞተችበት/ባት \|  \| \| --- \| --- \| |
| 104 | የትምህርት ደረጃ | 1. መደበኛ ትምህርት ያልተከታተለ/ች 2. አንደኛ ደረጃ የተማረ/ች(1-8) 3. ሁለተኛ ደረጃ የተማረ/ች(9-12) 4. ከፍተኛ ትምህርት የተከታተለ/ች |
| 105 | የመኖሪያ ቦታ | 1. ከተማ 2. ገጠር |
| 106 | ስራ | \| 1. የመንግሰት ሰራተኛ 2. የመንግሰት ሰራተኛ ያልሆነ/ች 3. ገበሬ 4. ራስ አስተዳዳሪ 5. የቤት እመቤት 6. ጡረታ የወጣ/ች \|  \| \| --- \| --- \| \| 1. ስራ የሌለው/ላት 2. ሌላ ከሆነ ይግለጹ------------ \| |

**Amharic version of the data collection tool (Questionnaire and checklist)**

| **ክፍል ሁለት፡ ትምባሆ መጠቀም፣ አልኮል መጠጣት እና የአካል ብቃት እንቅስቃሴ ሁኔታን የሚያሳይ መጠይቅ** | | | | **ወዴ ሌላ ጥያቄ**  **ለመሄድ** |
| --- | --- | --- | --- | --- |
| **ትምባሆ መጠቀም** | | | |  |
| 201 | እንደ ሲጋራ፣ሺሻ፣ ወይም የሚታኘክ ትምባሆ ያሉ የትምባሆ ምርቶችን ተጠቅመው ያውቃሉ? | 1. አዎ 2. አይደለም | | መልሶ አይደለም ከሆነ ወደ 204 ይሂዱ |
| 202 | በአሁኑ ጊዜ የትምባሆ ምርቶችን ይጠቀማሉ? | 1. አዎ 2. አይደለም | | መልሶ አይደለም ከሆነ ወደ 204 ይሂዱ |
| 203 | በአማካይ፣ በቀን ከሚከተሉት የትምባሆ ምርቶች ውስጥ ምን ያህል ይጠቀማሉ? | \| 1. ሲጋራ _------------_/ ቀን \| \| --- \| \| 1. የሺሻ ክፍለ ጊዜዎች ብዛት -------/ቀን \| \| 1. ሌላ ከሆነ (እባክዎ ይግለጹ) -----------------------/ ቀን \| \| | | |
| **አልኮል መጠጣት** | | | | |
| 204 | እንደ ቢራ፣ ወይን፣ ጠጅ፣ ጠላ፣ አረቄ፣ ኮረፌ...የመሳሰሉ የአልኮል መጠጦችን ጠጥተው ያውቃሉ? | 1. አዎ 2. አይደለም | | መልሶ አይደለም ከሆነ ወደ 207 ይሂዱ |
| 205 | አዎ ከሆነ፣ በአሁኑ ጊዜ አልኮል ይጠጣሉ? | 1. አዎ 2. አይደለም | | መልሶ አይደለም ከሆነ ወደ 207 ይሂዱ |
| 206 | በሳምንት/ወር ውስጥ ቢያንስ አንድ መደበኛ የአልኮል መጠጥ ስንት ቀን ይጠጣሉ? | \| 1. 1-2 ቀናት በሳምንት 2. 3-4 ቀናት በሳምንት 3. 5-6 ቀናት በሳምንት 4. በየቀኑ 5. 1-3 ቀናት በወር 6. ሌላ ከሆነ እባክዎ ይግለጹ**---------------** \| \| --- \| | | |
| **የአካል ብቃት እንቅስቃሴ** | | | | |
| 207 | ስራዎ ቢያንስ ለ10 ደቂቃዎች ያለማቋረጥ ከፍተኛ የአተነፋፈስ ወይም የልብ ምት እንዲጨምር የሚያደርግ **ጠንካራ እንቅስቃሴን** ያካትታል? እንደ ከባድ ሸክሞችን መሸከም ወይም ማንሳት፣መቆፈር ወይም የግንባታ ስራ | | 1. አዎ 2. አይደለም | መልሶ አይደለም ከሆነ ወደ 209 ይሂዱ |
| 208 | በሳምንት ውስጥ፣ ለስንት ቀናት ከፍተኛ የአተነፋፈስ ወይም የልብ ምት እንዲጨምር የሚያደርጉ **ብርቱ-የጥንካሬ** እንቅስቃሴዎችን ይሰራሉ? እንደ ከባድ ሸክሞችን መሸከም ወይም ማንሳት ፣ መቆፈር ፣ እርሻ ወይም የግንባታ ስራ | | የቀኖች ብዛት **___________** |  |
| 209 | ስራዎ ቢያንስ ለ10 ደቂቃዎች ያለማቋረጥ አነስተኛ የአተነፋፈስ ወይም የልብ ምት እንዲጨምር የሚያደርግ **መጠነኛ-ጥንካሬ** እንቅስቃሴን ያካትታል? እንደ ፅዳት፣ ፈጣን እርምጃ ወይም ቀላል ሸክም | | 1. አዎ 2. አይደለም | መልሶ አይደለም ከሆነ ወደ 211 ይሂዱ |
| 210 | በሳምንት ውስጥ፣ ለስንት ቀናት አነስተኛ የአተነፋፈስ ወይም የልብ ምት እንዲጨምር የሚያደርጉ **መጠነኛ የጥንካሬ** እንቅስቃሴዎችን ይሰራሉ? እንደ ፅዳት፣ ፈጣን እርምጃ ወይም ቀላል ሸክም | | የቀኖች ብዛት **___________** |  |
| 211 | ከቦታ ቦታ ለመንቀሳቀስ አብዛኛውን ጊዜ በእግር ይራመዳሉ ወይስ መጓጓዣ ይጠቀማሉ? | | 1. ብዙውን ጊዜ እራመዳለሁ 2. ብዙውን ጊዜ መጓጓዣ እጠቀማለሁ |  |
| 212 | በቀን ምን ያህል ጊዜ በመቀመጥ ያሳልፋሉ? | | ሰአታት_-----------------_ ደቂቃ_---------------_ |  |

| **ክፍል ሶስት: የአመጋገብ ሁኔታን የሚያሳይ መጠይቅ** | | | |
| --- | --- | --- | --- |
|  | ባለፉት 7 ቀናት ውስጥ ምን ያህል ቀን የሚከተሉትን ምግቦች ተመገቡ? | የቀኖች ብዛት | በጭራሽ |
| 301 | ማንኛውም አይነት የሰብል ምግቦች ፤በቆሎ፣ማሽላ፣ገብስ፣ ጤፍ፣ ስንዴ፣ሩዝ እና ከነዚህ የተሰሩ (ለምሳሌ ዳቦ፣እንጀራ፣ገንፎ፣ቂጣ፣ቆሎ)? |  |  |
| 302 | ማንኛውም አይነት ጥራጥሬ(ባቄላ፤አኩሪ አተር፤ሽምብራ፤ምስር፤ እና ከነዚህ የተሰሩ ? |  |  |
| 303 | ማንኛውም አይነት አትክልት፤ ጎመን፣ ሰላጣ፣ ቲማቲም ፣ ካሮት ወዘተ…? |  |  |
| 304 | ማንኛውም አይነት ፍራፍሬ፤ ሙዝ፤ብርቱካን፤አፕል ወዘተ…? |  |  |
| 305 | ማንኛውም አይነት ሥጋ፤ የበሬ፤የበግ፤የፍየል፤የዶሮ፤ አሳ፤ጉበት፤ ኩላሊት እና ልብ? |  |  |
| 306 | ማንኛውም አይነት እንቁላል? |  |  |
| 307 | ማንኛውም አይነት የወተት ተዋጽኦ ፤ ወተት፤አይብ እና እርጎ እና ሌሎች? |  |  |
| 308 | ማንኛውም አይነት ስኳር፤ማር? |  |  |
| 309 | ማንኛውም አይነት ዘይት፤ ቅቤ፤ በቅቤ የተሰራ ምግብ? |  |  |
| 310 | በቤትዎ ውስጥ ምግብ ለማብሰል ወይም ለማዘጋጀት ምን ያህል ጊዜ ጨው ይጨመራል? |  |  |
| 311 | ምን ያህል ጊዜ በፋብሪካ የተቀነባበሩ ምግቦችን ይመገባሉ? እንደ የታሸጉ ምግቦች, ጭማቂዎች፣ ለስላሳ መጠጦች …ወዘተ |  |  |

| **ክፍል አራት፡ የታካሚው/ዋ የአንትሮፖሜትር እና የክሊኒካል መረጃ** | | | **ወዴ ለላ ጥያቄ መሄድ** |
| --- | --- | --- | --- |
| 401 | ቁመት | ­­­­___________ሴንቲሜትር |  |
| 402 | ክብደት | ___________ኪሎ ግራም |  |
| 403 | ቢኤምአይ | ___________ኪ.ግ/ሜ^2^ |  |
| 404 | የወገብ ዙረያ ልኬት | ___________ሴንቲሜትር |  |
| 405 | የደም ግፊት መጠን | 1. ሲስቶሊክ የደም ግፊት   _________ሚ.ሜ ሜርኩሪ   1. ዲያስቶሊክ የደም ግፊት   _________ሚ.ሜ. ሜርኩሪ |  |
| 406 | የሜታቦሊክ ሲንድረም ህመም እንዳለብዎት ከታወቀ በኋላ ምን ያህል ጊዜ ሆኖት? | ----------ወር-----------ዐመት |  |
| 407 | ከቤተሰብዎ ውስጥ የሜታቦሊክ ሲንድረም ወይም የሜታቦሊክ ሲንድረም ክፍሎች (ስኳር፣ግፊት፣ስብ ክምችት ወይም የሆድ አካባቢ ውፍረት) ታሪክ ያለው ከእርስዎ ውጭ ሌላ ሰው አለ? | 1. አዎ 2. አይደለም | መልሶ አይደለም ከሆነ ወደ 409 ይሂዱ |
| 408 | ከሚከተሉት የሜታቦሊክ ሲንድሮም ክፍሎች ውስጥ የቤተሰብዎ አባል ያለው የትኛው ነው? (ከአንድ በላይ አማራጭ መምረጥ ይቻላል) | 1. የስኳር ህመም 2. የግፊት ህመም 3. የስብ ክምችት ወይም የኮሌስትሮል ህመም 4. የሆድ አካባቢ ውፍረት ህመም |  |
| 409 | ታካሚው/ዋ ለሜታቦሊክ ሲንድሮም ወይም ለሜታቦሊክ ሲንድሮም ክፍሎች የሚሆን በሀኪም ወይም በሌላ የጤና ባለሙያ የታዘዘ መድሀኒት ይወስዳል/ትወስዳለች? | 1. አዎ 2. አይደለም | መልሶ አይደለም ከሆነ ወደ 501 ይሂዱ |
| 410 | ምን አይነት መድሀኒት ነው ታካሚው/ዋ የሚወስደው/የምትወስደው?(ከአንድ በላይ አማራጭ መምረጥ ይቻላል) | 1. የግፊት ህመም መድሀኒት 2. የስኳር ህመም መድሀኒት 3. የስብ ክምችት ወይም የኮሌስትሮል ህመም መድሀኒት |  |
