# Supplementary material for: Hematological abnormalities and associated factors among metabolic syndrome patients at the University of Gondar comprehensive specialized hospital, Northwest Ethiopia
Source: PLoS One. 2023 May 25;18(5):e0286163. doi: 10.1371/journal.pone.0286163 (PMC10212162; doi:10.1371/journal.pone.0286163)
Supplement: Attachment — Submitted filename: Response to Reviewers.docx [file pone.0286163.s004.docx]

Date: April 13,2023

To: Plos One Journal

Subject: Sending a rebuttal letter

Dear Sir/madam, Greetings!

These is a rebuttal letter that responds to each point raised by the academic editor and reviewer(s).

For the Academic Editor

1. We ensure that our manuscript meets PLOS ONE's style requirements, including those for file naming.
2. We ensure that we removed any funding-related text from the manuscript and we agreed with the current Funding Statement which reads as follows: "The study was funded by the University of Gondar. The funders had no role in study design, data collection and analysis, decision to publish, or preparation of the manuscript." Though we have no update on our current funding statement, we included the above statement within our cover letter.
3. We included captions for our Supporting Information files at the end of our manuscript, and we updated any in-text citations to match accordingly.
4. We reviewed our reference list and we ensured that it is complete and correct.

For Reviewer 1

- You have recommended to add other common hematological abnormalities ….but as we are in resource limited country Ethiopia, we had financial, reagent as well as equipment constraints to add other hematology related measurements. But in the future if we got this supports we may certainly measure other hematology related parameters among metabolic syndrome patients, and we just put recommendations forward to other researchers to conduct more extensive studies.
- Abstract section: we try to mention who are patients of metabolic syndrome and how they are diagnosed, but the word limit for the abstract will exceed more than 300 words and this will be against the submission guidelines of the journal.
- Introduction part Line 124, we believe that including many types of hematological abnormalities will make the study more sound, rather than including only the common ones.
- Sampling methods says systematic random sampling: It is good to explain the selection processes with actual numbers !!

Answer: we explain the selection process with actual numbers, which can be read as follows: “A systematic random sampling technique was applied to select study participants at a regular interval (K) of two”.

- Line 246 : Authors did not mention the time when was FBS, TG, and HDL-C testes were done if there are time variation it could have impact on the interpretation of the results unless it is stated clearly including the limitation of secondary data . Moreover try to consider method variation, do all samples analyzed with similar instruments / methods?

Answer: it says that “Biochemical profiles such as FBS, TG, and HDL-C results on the date of data collection”.so these parameters were collected on the date of the data collection. For every answer: Mets patients these tests are inevitable tests when they come for checkup and we just collect these updated and timely results on the date of the data collection. Regarding the method variation, all biochemical tests were done with the same chemistry machine and since we only take the data from the patients’ charts we believe that we don’t need to mention the way these biochemical profiles were done.

- Line 276-277 : authors stated that “The automated hematology analyzer's performance was evaluated by running quality control reagents” I think here we need to check with known quality control materials it is not mentioned in your QA work.

Answer: It is obvious that quality control reagents are known quality control materials, and we mentioned that “The automated hematology analyzer's performance was evaluated by running quality control reagents (known quality control materials)”

- On table 3, please describe the stage of the parasites for Girdia lamblia and Entamoeba histolytica/diaper

All were trophozoite stages of e.histolytica and g.lamblia

- Line 363-364 : authors mentioned altitudes and smoking adjustment Hemoglobin value is described but how the adjustment was made is not mentioned in the methods section. It is good to state that to replicate the work.

Answer: We just put these on references as well as on the supporting informations.

Try to indicate anemia and other hematological abnormalities are happened before Mets or during Mets follow up?

Answer: Because of its cross-sectional nature, this study was unable to identify any causal relationships, and unable to indicate anemia and other hematological abnormalities are happened before Mets or during Mets follow up.

Sincerely!

**Amanuel Kelem**: Department of Medical Laboratory Sciences, Asrat Woldeyes Health Science Campus, Debre Berhan University, Debre Berhan, Ethiopia. **E-mail**: amanuelkelem@gmail.com. Tel.: +251-9-11712112

**Elias Shiferaw**: Department of Hematology and Immunohematology, School of Biomedical and Laboratory Sciences, College of Medicine and Health Sciences, University of Gondar, Gondar, Ethiopia. **E-mail**: [eliasshiferaw2008@gmail.com](mailto:eliasshiferaw2008@gmail.com). Tel.: +251-9-27607075

**Tiruneh Adane**: Department of Hematology and Immunohematology, School of Biomedical and Laboratory Sciences, College of Medicine and Health Sciences, University of Gondar, Gondar, Ethiopia. **E-mail**: [tirunehadane01@gmail.com](mailto:tirunehadane01@gmail.com). Tel.: +251-9-18754325
